# Morphology and carrier non-geminate recombination dynamics regulated by solvent additive in polymer/fullerene solar cells†

**DOI:** 10.1039/d0ra03389h

**Published:** 2020-06-17

**Authors:** Ming-Ming Huo, Rong Hu, Qing-Shan Zhang, Shaoting Chen, Xing Gao, Yi Zhang, Wei Yan, Yong Wang

**Affiliations:** Laser Institute, Qilu University of Technology (Shandong Academy of Sciences) Qingdao Shandong 266100 China yongwang@sdlaser.cn mingminghuo@sdlaser.cn shaoting.chen@sdlaser.cn weiyan@sdlaser.cn; Research Institute for New Materials Technology, Chongqing University of Arts and Sciences Chongqing 402160 China hurong_82@cqwu.edu.cn 2584435203@qq.com 1345542490@qq.com 2803904178@qq.com

## Abstract

In this study, PBDTTT-E (based on benzo [1,2-b:4,5-b′] dithiophene (BDT) and thieno [3,4-b] thiophene (TT)) as a donor and fullerene derivative PC_71_BM (phenyl-C_71_-butyric acid methyl ester) as an acceptor with and without 1,8-diiodooctane (DIO)-treated copolymer solar cells were investigated. The device based on PBDTTT-E with treated DIO showed remarkably high current density (*J*_sc_), fill factor (FF) and similar open-circuit voltage (*V*_oc_). Charge carrier lifetime (*τ*_*n*_), density (*n*) and non-geminate recombination rate (*k*_rec_) in the photoactive layers were measured by employing transient photovoltage (TPV) and charge extraction (CE) techniques. Based on *k*_rec_ and *n*, *J*–*V* curves were reconstructed. The DIO optimized the morphology of the active layer and its PBDTTT-E:PC_71_BM interfaces were increased. Therefore, compared to the device without the treated DIO, the device with the treated DIO showed larger electron mobility, longer carrier lifetime (*τ*_*n*_) and lower non-geminate recombination rate (*k*_rec_), which enhances the carrier transport and restrains the non-geminate recombination, realizing the higher *J*_sc_ and FF. In addition, that the DIO-treated devices can weaken the role of other factors (such as field dependent geminate recombination) in limiting device performance. The results provide some hints of improved device performance upon DIO as an additive in the D–A type polymer/fullerene solar cells.

## Abstract 2

The device (PBDTTT-E:PC_71_BM) with DIO treated show lower non-geminate combination rate (*k*_rec_) and non-geminate combination current (*J*_NGR_). This indicates that DIO treatment can restrain the non-geminate recombination, realizing the higher *J*_sc_ and FF.

## Graphical abstract

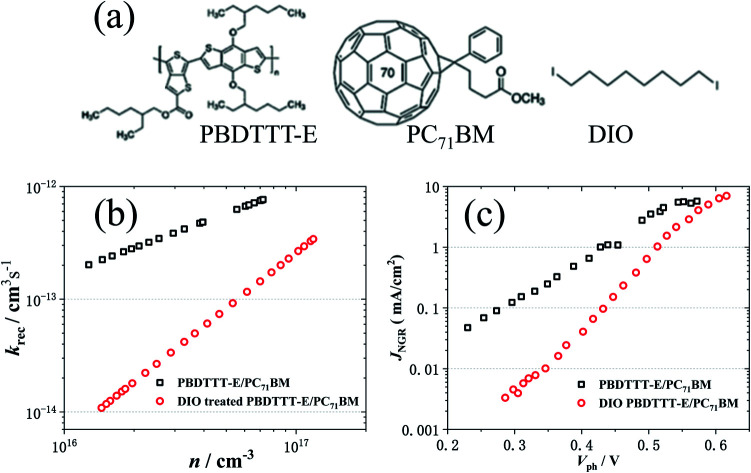

## 1. Introduction

Polymer solar cells (PSCs) are attracting extensive interest for their potential low cost, light-weight and solution-processed large-scale fabrication with power conversion efficiencies (PCEs) now exceeding 15% in labs.^1,2^ Improvements in the performance of polymer solar cells will be accelerated by a better understanding of the physical processes involved in device operation.^3^ A key consideration for evaluating the limitations on device efficiency is the extent to which this increase in the charge density results in the acceleration of charge carrier loss pathways. Such loss pathways, including in particular non-geminate recombination, may limit the collection of photogenerated charges by the device electrodes. However, the quantification of the magnitude of non-geminate recombination losses in PSCs remains controversial.^4^ Some reports believed that the non-geminate recombination is unimportant in PSCs because the non-geminate recombination coefficient is several orders of magnitude smaller than predicted using a Langevin description.^5,6^

Transient photovoltage (TPV) and charge extraction (CE) have been used to investigate non-geminate recombination dynamics to better understand their role in determining the power conversion efficiency of devices. TPV and CE techniques are performed under standard device PV operating conditions in terms of light intensity.^7^ They were first applied in dye-sensitized solar cells (DSSCs), as described by O'Regan *et al.*^8^ and Peter *et al.*^9^ and was subsequently adapted by Shuttle *et al.*^7^ to determine the charge carrier decay in P3HT/PC_61_BM solar cells. Now, TPV and CE techniques have been widely used in the systems of fullerene and non-fullerene acceptor PSCs, quantum dot (QD)-based solar cells, hybrid perovskite solar cells and so on. With the help of the two techniques, it has been worked out that the *V*_oc_ and FF of PSC devices are primarily limited by non-geminate recombination^4,10,11^ and dark current originates from non-geminate recombination at the polymer/fullerene interface.^12^ Hence, TPV and CE techniques are effective tools for achieving the systematic optimization of the voltage output of organic photovoltaic devices.

One factor constraining the PCEs of bulk heterojunction PSC device is the morphology of the interpenetrating networks of donor and acceptor materials in the photoactive layer.^13^ The network must have multiple interfaces for efficient charge separation and long pathways for efficient charge transfer to achieve high PCEs.^14,15^ Such morphologies can be achieved by applying numerous methods, including postproduction thermal annealing,^16^ solvent vapor annealing,^17^ polymer configuration optimization,^18^ and the use of solvent additives.^13,19^ Among these methods, a classical additive agent, namely 1,8-diiodooctane (DIO), with a concentration range of 0.5–3%, can optimize the morphology of active layers and enhance the performance of PSCs effectively. Many evidences have demonstrated that the introduction of DIO can decrease the polymer domain size and increase exciton dissociation efficiency at the D–A interface. For example, B. A. Collins *et al.* studied the role of additives on the nanoscale domain size, distribution and composition in PTB7:PC_71_BM devices *via* resonant X-ray scattering and microscopy. They concluded that DIO dramatically shrinks the domain size of pure fullerene agglomerates that are embedded in a polymer-rich 70/30 wt% molecularly mixed matrix while preserving the domain composition relative to additive-free devices. The increased domain interface is primarily responsible for the dramatic increase in device performance.^20^ N. Jain *et al.* believed that the use of DIO can have differing effects on bulk and interfacial intermolecular ordering in devices. In PTB7:PC_71_BM system, there is a favorable steric interactions between polymer and fullerene (the interfacial properties are least affected by DIO treatment), enabling improvements in *J*_sc_ and FF to be enjoyed without compromising on *V*_oc_. In the PCPDTBT:PC_71_BM system, the use of DIO resulted in an increase in interfacial disorder (interfacial traps between D and A domains), which limited the achievable *V*_oc_.^21–23^ However, most of the current research works are focused on the effects of DIO treatment, the relationship between the morphology of active layers and apparent performance of the PSC devices but the underlying process of carrier recombination dynamics particularly non-geminate recombination are not yet fully understood in the DIO-treated PSC device.

In this study, charge carrier lifetime, density and non-geminate recombination rate of PBDTTT-E:PC_71_BM solar cells with and without the treatment of DIO were studied to explore the origin of influenced performance *via* the TPV and CE techniques. The data from TPV transient measurements can provide information about the transport and recombination of charge carriers in a device. The CE data information can acquire the charge concentration stored in the polymer/fullerene heterojunction. The results indicated that the device with treated DIO had more D–A phase interfaces in morphology characteristics, which were beneficial for the generation and transfer of carriers, resulting in long carrier lifetime and low bimolecular combination rate (*k*_rec_). These features suggest that the DIO treatment helps to optimize the morphology and enhance carrier transport. The results provide some hints to understand the direct relevance among the morphology of the additive-controlled active layer, carrier recombination dynamics and performance of devices.

## 2. Materials and methods

### Film and device preparation

PBDTTT-E and PC_71_BM were purchased from Solarmer Inc (Beijing). The structure of PSCs was constructed by standard inverted configuration based on the method of literature,^24,25^*i.e.*, indium tin oxide (ITO) substrate/zinc oxide (ZnO)/photoactive layer/molybdenum oxide (MoO_3_)/Ag electrode. The indium tin oxide (ITO) substrate was successively cleaned by detergent, deionized water, acetone, ethanol and isopropyl alcohol, and then dried in a dry heat oven. To obtain the ZnO electron transport layer, 60 μL of the precursor solution, which contained zinc acetate : 2-methoxyethanol : ethanolamine (1 g : 10 mL : 0.28 mL) was spin-coated (3000 rpm, 30 s) on the ITO substrate, followed by annealing for 1 hour on the heating plate at 200 °C. The precursor solutions of active layers were the PBDTTT-E : PC_71_BM solution (9 mg mL^−1^ : 13.5 mg mL^−1^, CB), PBDTTT-E : PC_71_BM solution (9 mg mL^−1^ : 13.5 mg mL^−1^, CB : DIO = 97% : 3%, by volume), respectively. The preparations of active layers were conducted in a glove box filled with nitrogen. The thickness of the active layers was 90 nm. Finally, a thickness of 8 nm MoO_3_ hole transport layer and 50 nm Ag electrodes were successively deposited on the surface of the active layer using a shadow mask to obtain the effective area (0.07 cm^2^) of the devices.^26^

### For TPV measurements

The target PSC device was kept under open circuit conditions and was irradiated by a 532 nm continuous laser to maintain a desired photovoltage (*V*_ph_). Placing neutral density filters into the illumination path caused a systematic change under the initial quasi-equilibrium conditions. A perturbation pulse laser (10 ns, 3 Hz) was then applied and was overlapped with the continuous spot in space to induce a small increase in *V*_ph_, (Δ*V*_ph_/*V*_ph_ ≈ 5%). The electric signals were recorded by an oscilloscope.^27^ The TPV transients studied herein were fitted to a single exponential function as eqn (1) in order to calculate the small perturbation carrier decay time *τ*_Δ*n*_:

1 Δ*V* = Δ*V*_0_e^−*t*/*τ*_Δ*n*_^

where *t* is the time, Δ*V* is the increase in voltage, Δ*V*_0_ is the transient amplitude at *t* = 0, and *τ*_Δ*n*_ is the lifetime of the transient, respectively.

As it has been previously discussed by Durrant *et al.*,^11,28^ the small perturbation lifetime *τ*_Δ*n*_ can be related to the overall carrier decay time by eqn (2):

2 *τ*_*n*_ = (*λ* + 1)*τ*_Δ*n*_

where *λ* is the experimentally determined constant, (*λ* + 1) corresponds to the overall reaction order. Because the primary reservoir of recombining charge in PSC at open-circuit is located within the bulk heterojunction, the loss pathway measured by the TPV technique corresponds primarily to that affecting charge in the active layer.^29^

### For CE measurements

Illumination was provided by a 532 nm continuous laser. The *V*_ph_ of target PSC was varied by varying the laser intensity. The laser was typically turned on for approximately 100 ms in order to allow the device to reach steady state conditions without overheating. The laser was switched off and the PSC was short-circuited using a fast switch unit (switch times < 100 ns, internal resistance ∼ 130 Ω). The voltage transients were recorded by the oscilloscope. These voltage transients were converted into current transients using Ohms law (current transient = voltage transient/internal resistance). The current transients were integrated with respect to time to calculate the charge quantity (*q*) in the cell. Then, the total charge density (*n*) was calculated from eqn (3):

3 *n*(*V*_ph_) = *q*(*V*_ph_)/*edA*

where *e* is the elementary charge, *d* is the thickness of the photoactive layer of the device, and *A* is the area of the device.

## 3. Results and discussion

**Fig. 1:**
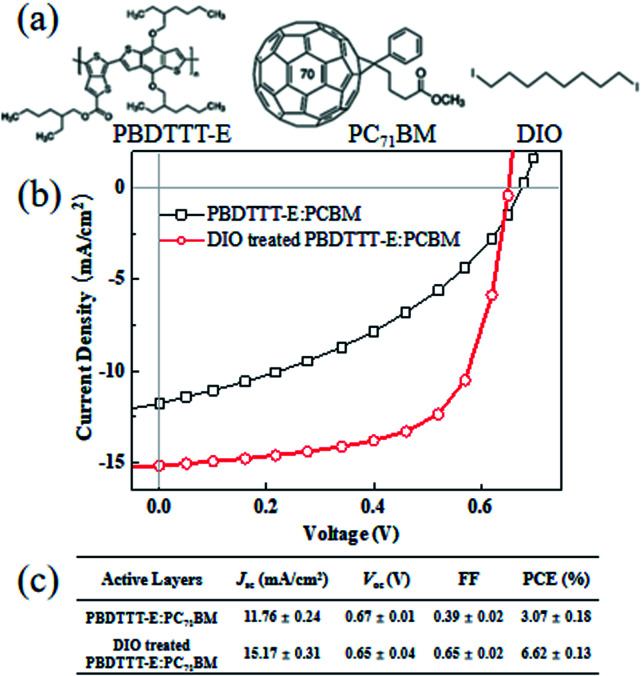
(a) Chemical structures of PBDTTT-E, PC_71_BM and DIO; (b) *J*–*V* of PBDTTT-E:PC_71_BM and DIO-treated PBDTTT-E:PC_71_BM devices. (c) The performance parameters of each device.

The chemical structures of donor PBDTTT-E, acceptor PC_71_BM and additive DIO in this study are shown in Fig. 1a. The typical *J*/*V* graphs and performance parameters of PBDTTT-E:PC_71_BM devices based on DIO treated and without DIO treated under the illumination of an AM 1.5 G solar simulator (100 mW cm^−2^) are shown in Fig. 1b and c, respectively. The device without DIO treated shows an open-circuit voltage (*V*_oc_) of 0.67 V, a short-circuit current density (*J*_sc_) of 11.76 mA cm^−2^ and a fill factor (FF) of 39%, producing a power conversion efficiency of 3.07%. In contrast, the device based on DIO treated shows a similar *V*_oc_ of 0.65 V, a significantly increased *J*_sc_ of 15.17 mA cm^−2^ and FF of 65%, resulting in a PCE of 6.62%. The similar *V*_oc_ and enhancement of *J*_sc_ and FF are consistent with Jain's report on PTB7:PC_71_BM devices.^21^ There is a favorable steric interaction between PBDTTT-E and fullerene (the interfacial properties are least affected by DIO treatment), enabling improvements in *J*_sc_ and FF to be enjoyed without compromising *V*_oc_.^21^ The improvements in *J*_sc_ and FF are very closely related to carrier transients for the devices with DIO processing. Here, the DIO content that we mentioned in this study have been optimized.

**Fig. 2:**
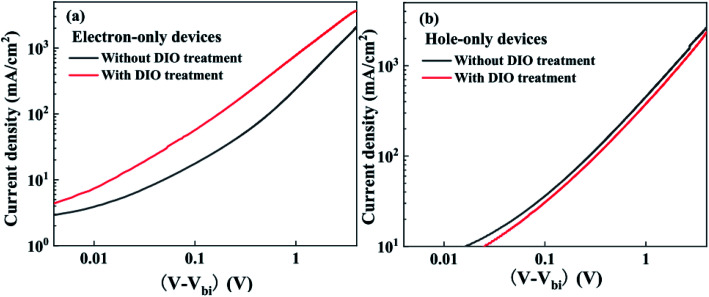
The *J*–*V* characteristics of electron-only (a) and hole-only (b) devices under dark conditions.

The mobility of hole and electron for corresponding devices with and without DIO treated was determined using the space-charge-limited current (SCLC) method.^30^ The hole-only devices with a configuration of ITO/MoO_3_/active layer/MoO_3_/Ag and electron-only devices with a configuration of ITO/ZnO/active layer/LiF/Al were fabricated. The hole mobility in the hole-only devices and the electron mobility in the electron-only devices can be calculated using the Motte–Gurney law as eqn (4).4
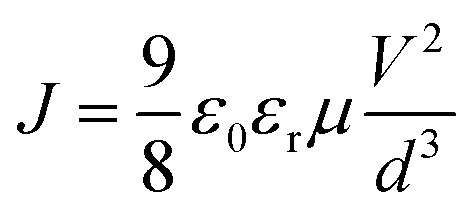
where *ε*_r_ is the relative permittivity of polymer assumed to be 3, which is a typical value for conjugated polymers. *ε*_0_ is the vacuum dielectric constant of 8.85 × 10^−12^ F m^−1^. *V* is the voltage, and *d* ≈ 90 nm is the thickness of the active layer. The *J*–*V* characteristics of single carrier devices are shown in the Fig. 2, and the extracted values of mobility are listed in Table S1.† The zero field mobility (*μ*_0_), the field dependent parameter (*β*_F_) and mobility (*μ*) as a function of applied electric field (*E*) were calculated in part 3 of ESI.† The hole and electron mobility of the PBDTTT-E:PC_71_BM device are similar with each other with values of 5.818 × 10^−4^ cm^2^ V^−1^ S^−1^ and 4.425 × 10^−4^ cm^2^ V^−1^ S^−1^, respectively. The hole mobility of the DIO-treated PBDTTT-E:PC_71_BM device is similar with the values of mobility (the hole and electron) of the PBDTTT-E:PC_71_BM device, that is, 4.910 × 10^−4^ cm^2^ V^−1^ S^−1^. The electron mobility of the DIO-treated PBDTTT-E:PC_71_BM device is larger than that of others, that is, 7.723 × 10^−4^ cm^2^ V^−1^ S^−1^. Our results are consistent with the report that the hole mobility is relatively insensitive to addition, but the electron mobility increased significantly upon the addition of the processing additive.^19^

**Fig. 3:**
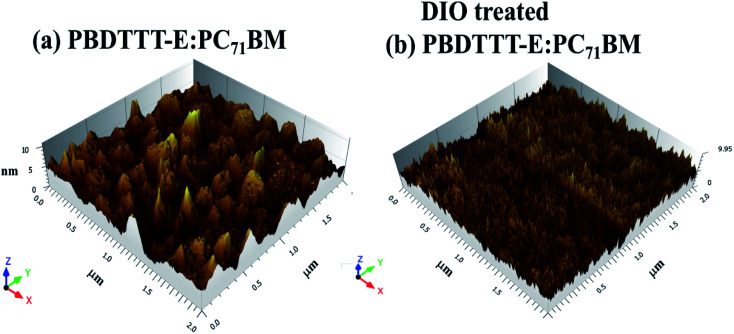
Three dimensional (3D) AFM images of (a) PBDTTT-E:PC_71_BM without DIO-treated and (b) PBDTTT-E:PC_71_BM with DIO-treated blend films.

To characterize morphology differences of the active layers in devices with and without the DIO treatment, AFM characterization was carried out using the tapping mode. The three dimensional (3D) AFM images of active layers are shown in Fig. 3. There are large domains in the active layer of PBDTTT-E:PC_71_BM without DIO treatment, which may diminish the exciton migration to the donor/acceptor interface and are not favorable for charge separation.^31,32^ The morphology of the active layer of PBDTTT-E:PC_71_BM with the DIO treatment is much more uniform and there is no large phase separation, showing good miscibility between PBDTTT-E, PC_71_BM and the formation of interpenetrating networks. It is reported in the PBDTTT-C:PCBM and PTB7:PCBM^13,20^ devices that DIO could selectively dissolve PC_71_BM aggregates, allowing their interrelation into polymer domains and thereby change the distribution of the domain size and increase the polymer: PC_71_BM interface. The more homogeneous PC_71_BM dispersion in the network of PBDTTT-E could be formed during the slow volatilization process of the DIO solvent, as a result of the improved morphology, the whole device performance increased due to significant enhancement of *J*_sc_ and FF.

**Fig. 4:**
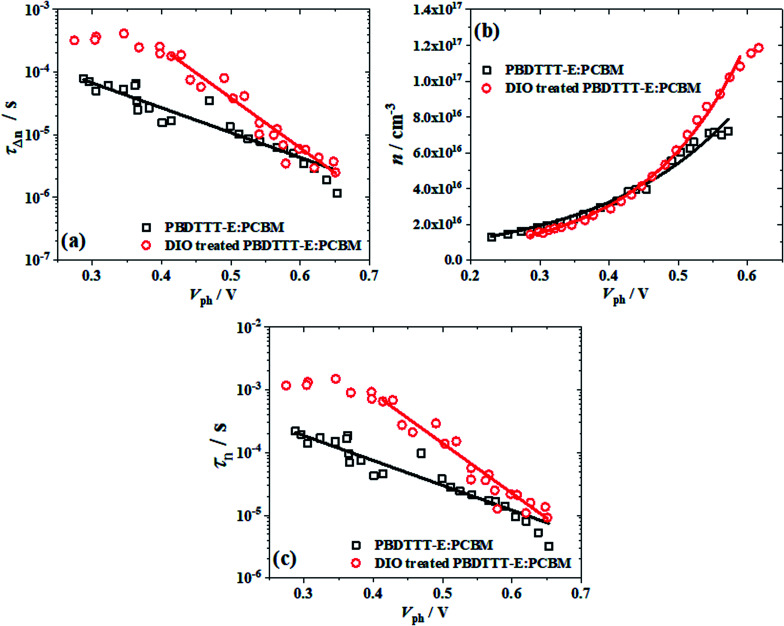
(a) The transient lifetime (*τ*_Δ*n*_), (b) charge carrier density (*n*) and (c) the overall carrier lifetime (*τ*_*n*_) as a function of the open circuit voltage (*V*_ph_), the black □ represents PBDTTT-E:PC_71_BM without treated DIO and the red ○ represents the DIO-treated PBDTTT-E:PC_71_BM devices. Lines represent the fit to each equation.

For a deeper understanding of how the changes in the morphology of active layers affect our device performance, the transient lifetime (*τ*_Δ*n*_) and charge carrier density (*n*) as a function of the open circuit voltage (*V*_ph_) were detected by employing the TPV and CE techniques to research carrier transients. The small perturbation transient lifetime (*τ*_Δ*n*_) for both devices can be obtained by fitting the transient photovoltage decays at different illumination intensities with an exponential decay course. Fig. 4a shows the *τ*_Δ*n*_ as a function of *V*_ph_ for PBDTTT-E:PC_71_BM with DIO treated and without DIO treated devices. *τ*_Δ*n*_ decreases as *V*_ph_ increases, according to eqn(5):

5 *_____τ_____*_Δ*n*_ = *τ*_Δ*n*_0__*e*^−*βV*_ph_^

where *τ*_Δ*n*_0__ is the transient carrier lifetime without illumination and *β* is the decay constant, respectively.^11^ Herein, the values of *τ*_Δ*n*_0__ and *β* are respectively determined as 0.37 s and 18.34 V^−1^ for DIO-treated PBDTTT-E:PC_71_BM solar cell, while their values changed to 0.001 s and 9.129 V^−1^ in the PBDTTT-E:PC_71_BM device. The larger *β* value means it has a more steep decay order.

Fig. 4b indicates the charge carrier density (*n*) as a function of *V*_ph_, which was measured using the charge extraction (CE) method. The *n* remains the same in the low voltage regions, which are mainly the electrode-dominated charge distributions.^29^ It is apparent that charge carrier density increases exponentially as a function of *V*_ph_, following a relationship below.

6 *n* = *n*_0_*e*^*γV*_ph_^

where *n*_0_ is the charge carrier density under the ark and *γ* is the slope of ln(*n*)–*V*_ph_ curves.^11^ Therefore, the fitted *n*_0_ is ∼1.90 × 10^15^ cm^−3^ and *γ* is ∼6.95 V^−1^ for PBDTTT-E:PC_71_BM with the DIO-treated device, while *n*_0_ is ∼4.11 × 10^15^ cm^−3^ and *γ* is ∼5.16 V^−1^ for the PBDTTT-E:PC_71_BM cell without DIO. The *n*(*V*_ph_) curve visualizes the intrinsic property of a semiconductor. Eqn (6) expresses that the carrier density (*n*) in active layers is dependent upon a certain bulk quasi-Fermi level splitting and is therefore representing an effective electronic bandgap.^11,33^ The value of *γ* is less than the value expected when non-geminate recombination occurs in a medium with no traps (which would correspond to *γ* < *e*/2*k*_B_*T* = ∼19 V^−1^).^10^ This non-ideal behavior is assigned to the presence of an exponential tail of states extending into the bandgap of the photoactive layer.^4^ These traps correspond to the presence of energetic disorders and influence both transport and recombination.^34^ Smaller *γ* means that the device has a higher degree of trap states within the photoactive layer.^35^ Consequently, compared with the PBDTTT-E:PC_71_BM device, the DIO-treated PBDTTT-E:PC_71_BM has a lower degree of trap states. The role of the DIO additive decreases the trap state degree of the active layer during the film-forming process.

Having obtained the relationship between *τ*_Δ*n*_ and *V*_ph_ as well as the behavior of *n* with *V*_ph_, the overall order of reaction can be determined, as defined by eqn (7).^11^7
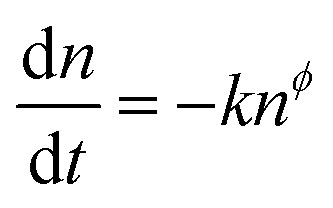
where *ϕ* = *λ* + 1 is the overall reaction order for charge carrier losses with respect to the charge density, *k* is the rate coefficient for the charge carrier decay. The *ϕ* can be obtained directly from parameters *β* and *γ* of eqn (4) and (5), that is, eqn (8), or the data shown in Fig. 4(a) and (b) can be combined to plot *τ*_Δ*n*_*versus n* in Fig. S1,† from Fig. S1,† we can obtain the value *λ*.8
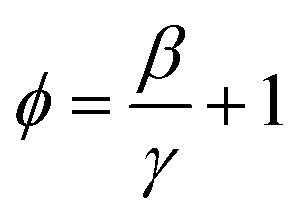


Therefore, we can get *λ* = ∼2.640 and 1.769 for PBDTTT-E:PC_71_BM with and without DIO treatment devices, respectively. According to Fig. 1, eqn (2) and the value of *λ*, the overall carrier decay time *τ*_*n*_ can be obtained, which is shown in Fig. 4c. Hence, *τ*_*n*_ of the DIO-treated devices are larger than that of PBDTTT-E:PC_71_BM without DIO-treated devices. Combining the parameters *λ*, *n*_0_, *τ*_Δ*n*_0__ and *n*, the effective nongeminate recombination coefficient (*k*_rec_) can be calculated from the TPV/CE data by eqn (9),^11^9
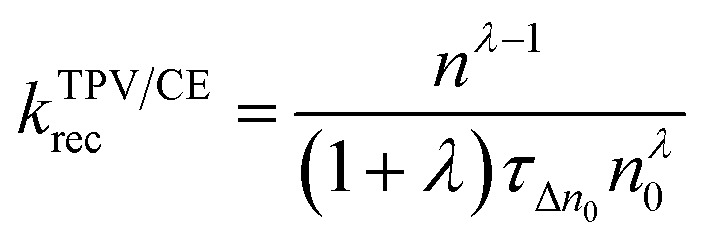


**Fig. 5:**
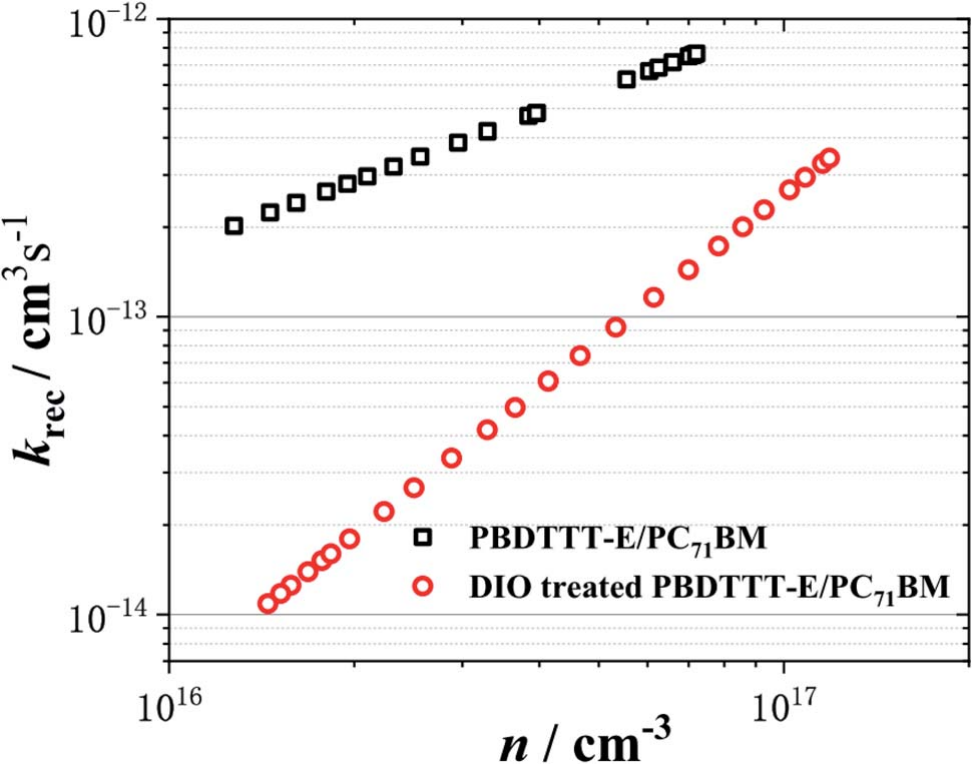
The non-geminate recombination coefficient (*k*_rec_) as a function of the charge carrier density (*n*). The black □ and red ○ represent the PBDTTT-E:PC_71_BM without and with DIO-treated devices, respectively.

The characteristic curves of carrier density *versus k*_rec_ is plotted, as shown in Fig. 5. Apparently, the *k*_rec_ is dependent on the charge density. This charge density dependence can be understood as being derived from the presence of trap states in the photoactive layer of the film. As the charge density is increased, the deepest traps were filled, resulting in an increased average charge carrier mobility.^11^

In all cases of Fig. 5, *k*_rec_ shows a power law feature. It is important for a PSC device function that *k*_rec_ increases with *n*, as it results in a strongly nonlinear increase in non-geminate recombination losses with increasing charge density. It is apparent that the PBDTTT-E:PC_71_BM with the DIO-treated device exhibits the lower *k*_rec,_ with its slower recombination dynamics, as shown in Fig. 4c. When compared at the same equivalent charge density of 3.0 × 10^16^ cm^−3^, the non-geminate recombination rate coefficient for the device with the DIO treatment is an order of magnitude lower than that for the device without DIO treatment. Owing to slower non-geminate recombination losses, the device with DIO treated can afford higher charge accumulation. According to *k*_rec_, the Langevin reduction factor (*ζ*) can be determined, as shown in Fig. S2.† The reduction factor for the device with DIO treatment is an order of magnitude lower than that for the device without DIO treatment too, and it is suggested that the non-geminate recombination of the device with DIO treated is more suppressed and hence charge carriers can survive for a longer time. The lower *k*_rec_ and *ζ* can enhance the carrier transport and restrain the non-geminate recombination.

In order to quantify the impact of non-geminate recombination upon the device *J*–*V* curves, based on the relationship between *J*_NGR_ and *τ*, *n*:^35^10
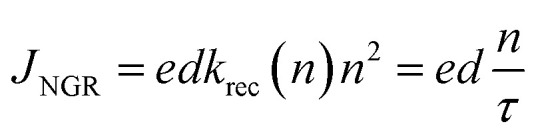


**Fig. 6:**
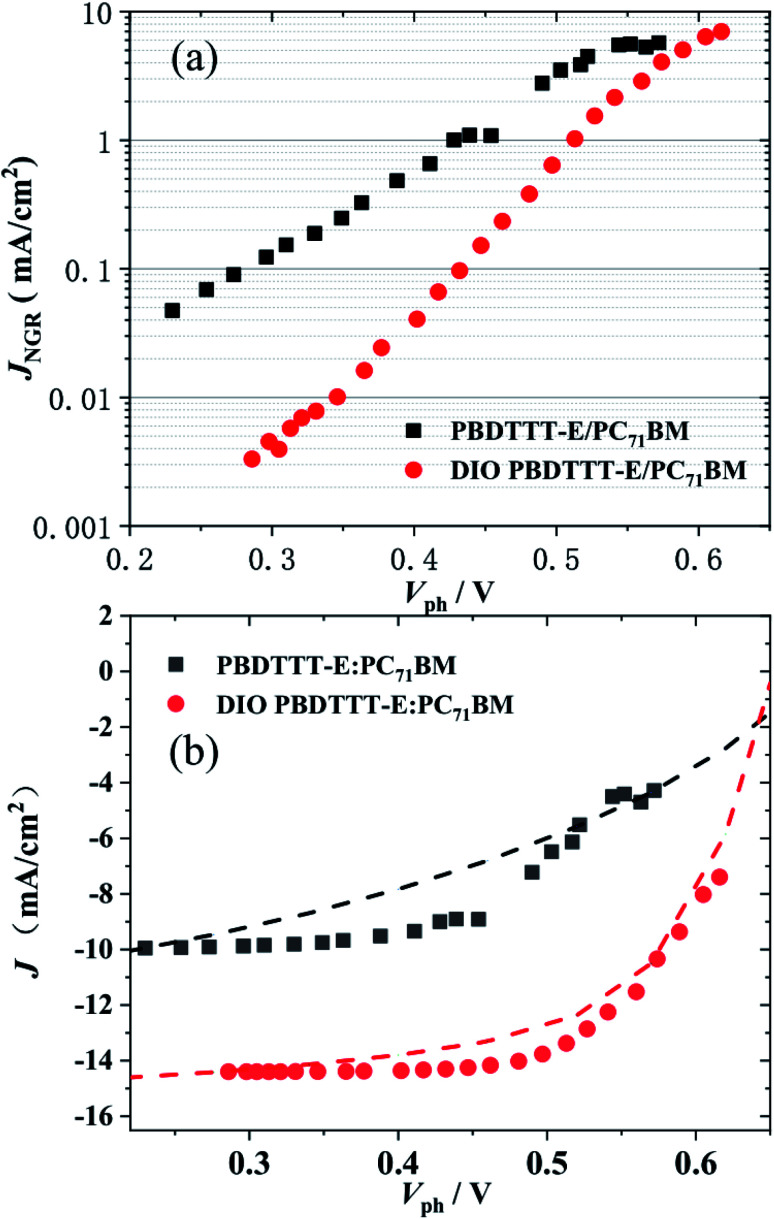
(a) The measured non-geminate recombination current density *J*_NGR_, under *V*_ph_. (b) Experimental *J*–*V* curves (dashed lines) under simulated AM 1.5 illumination. Compared with the calculated *J*–*V* data (points) for PBDTTT-E:PC_71_BM with (red ○) and without DIO (black □) treated.

The non-geminate recombination current density, *J*_NGR_, under *V*_ph_, for PBDTTT-E:PC_71_BM with and without DIO-treated devices were measured in Fig. 6a. The two devices show non-geminate recombination losses at open circuit of similar magnitude to their respective *J*_sc_, indicating that the dominant loss pathway limiting *V*_oc_ in all cases is non-geminate recombination.^36^ However as the device voltages reduce towards the near short circuit, there is a qualitative difference in the behaviour of the PBDTTT-E:PC_71_BM with a DIO-treated cell relative to the device without DIO. The magnitude of *J*_NGR_ near the short circuit in the PBDTTT-E:PC_71_BM without DIO-treated devices is much larger (>0.04 mA cm^−2^) than in the device with DIO-treated (∼0.003 mA cm^−2^). This indicates that the *J*_sc_ and FF for PBDTTT-E:PC_71_BM without a DIO device is limited by a non-geminate loss process.

Based on the eqn (11), *J*(*V*) can be calculated:

11 *J*_GEN_(*V*) = *J*(*V*) − *J*_NGR_(*n*,*V*)

Fig. 6b compares the experimentally measured *J*–*V* curves (dashed lines) with the reconstructed *J*–*V* curve (points). There are some discrepancies, which most likely result from the simplicity of our experimental techniques. For example, the illumination emanated from the 532 nm laser diode but not the white light and the devices treated with DIO were almost reproduced. As only non-geminate losses were considered to calculate the *J*/*V* behavior, they were identified as the dominant loss process responsible for device performance limitation. However, in the case of the device without DIO treated, there were some larger discrepancies in the region of 0.3–0.5 V. According to the report of A. Foertig *et al.*^37^ and G. F. A. Dibb *et al.*,^36^ other factors such as field-dependent geminate recombination also play an important role in limiting the performance of the device without the treatment of DIO.

Based on the above analysis, the effect of DIO on the morphology of the active layer of the PBDTTT-E:PC_71_BM system increases the PBDTTT-E:PC_71_BM interfaces and formation of interpenetrating networks. This advantage of morphology will facilitate charge separation and transfer. Therefore, the electron mobility increased significantly upon the addition of processing additives. According to the value of *γ* from *n*(*V*_ph_) curve, we conclude that DIO decreases the degree of trap state of the active layer. There are longer average carrier lifetimes (*τ*_*n*_), lower non-geminate recombination rate (*k*_rec_) and reduction factor (*ζ*) in the device with DIO treated. By calculating *J*_NGR_ and reconstructing the *J*–*V* curve, it can be concluded that the non-geminate loss process limits the *J*_sc_ and FF for the PBDTTT-E:PC_71_BM devices. DIO-treated devices can weaken the important role of other factors (such as field-dependent geminate recombination) in limiting device performance. All the above advantages of DIO-treated devices correspond with the morphology characteristics that interpenetrating networks of donors and acceptors observed in the active layer.

## 4. Conclusions

In summary, the non-geminate recombination dynamics of PBDTTT-E:PC_71_BM devices with and without the DIO treatment were studied through transient optoelectronic measurements. The study included the measurements of charge carrier lifetime (*τ*_*n*_), density (*n*), and non-geminate recombination rate (*k*_rec_) in the photoactive layer of device. The DIO treatment can increase the PBDTTT-E:PC_71_BM interfaces and formation of interpenetrating networks, which affect the performance of devices, mainly *J*_sc_ and FF. TPV and CE measurements indicated that there is a longer average carrier lifetime (*τ*_*n*_), lower non-geminate recombination rate (*k*_rec_) and reduction factor (*ζ*) in the device with the DIO treatment. In addition, the electron mobility increased significantly upon the addition of processing additive. Above all, this can enhance the carrier transport and restrain the non-geminate recombination. By calculating *J*_NGR_, it can be concluded that the non-geminate loss process limits the *J*_sc_ and FF for the PBDTTT-E:PC_71_BM devices. By reconstructing the *J*–*V* curve, it can be observed that DIO-treated devices weaken the important role of other factors (such as the field-dependent geminate recombination) in limiting device performance. The results provide important reference values for the fabrication of high performance PSC devices by employing solvent additive engineering.

## Conflicts of interest

The authors declare no competing financial interests.

## Supplementary Material

RA-010-D0RA03389H-s001
