# Supplementary material for: Morphology and carrier non-geminate recombination dynamics regulated by solvent additive in polymer/fullerene solar cells
Source: RSC Adv. 2020 Jun 17;10(39):23128–35. doi: 10.1039/d0ra03389h (PMC9054697; doi:10.1039/d0ra03389h)
Supplement: RA-010-D0RA03389H-s001 [file RA-010-D0RA03389H-s001.pdf]

Electronic Supplementary Material (ESI) for RSC Advances.
This journal is © The Royal Society of Chemistry 2020

Supplementary data

Morphology and Carrier Non-Geminate Recombination Dynamics Regulated by solvent additive in Polymer/Fullerene Solar Cells

Ming-Ming Huo[(a)], Rong Hu[(b), *], Qing-Shan Zhang[(b)], Shaoting Chen[(a)], Xing Gao[(b)], Yi Zhang[(b)], Wei Yan[(a)], Yong Wang[(a)]

(a)Laser Institute, Qilu University of Technology (Shandong Academy of Sciences), Qingdao, Shandong 266100, China.

(b)Research Institute for New Materials Technology, Chongqing University of arts and sciences, Chongqing 402160, China

1. The small perturbation charge carrier lifetime ($\tau_{\Delta n}$) as a function of charge carrier density (n) for PBDTTT-E:$PC_{71}BM$ with and without DIO treated. The solid line is from the fitting curves of Figures 4(a) and 4(b) in manuscript.

According to Figure 4(a) and 4(b) in manuscript, the power-law ($\tau_{\Delta n}=a\cdot n^{-\lambda}$, a is a constant) relationship between small perturbation carrier lifetime ($\tau_{\Delta n}$) versus the carrier concentration (n) have been shown in Figure S1. Too few data points may cause larger deviation, so the solid line can be fitted, for device without DIO is $\tau_{\Delta n}=4.3148\times10^{24}\cdot n^{-1.7688}$, for device with DIO treated is $\tau_{\Delta n}=2.8097\times10^{43}\cdot n^{-2.6390}$. The power ($\lambda$) is 1.7688 and 2.6390 for the device without and with DIO treated, respectively.

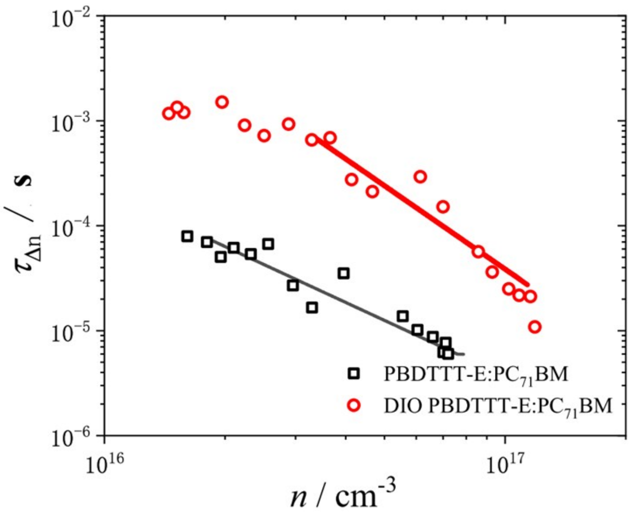

Figure S1: The small perturbation charge carrier lifetime ($\tau_{\Delta n}$) as a function of charge carrier density (n) for PBDTTT-E:$PC_{71}BM$ with (o red circle) and without (□ black square) DIO treated. The solid line is from the fitting curves of Figures 4(a) and 4(b) in manuscript.

2. The langevin reduction factor (ζ)

The Langevin reduction factor (ζ) can be determined by equation S1[S1]:

$$\zeta = \frac{k_{rec}}{k_L} \tag{S1}$$

Where k_{rec} is the non-geminate recombination coe□cient， k_L is the upper limit of the recombination coefficient, described by the langevin model (equation S2):

$$k_L = \frac{e}{\varepsilon\varepsilon_0}\left(\mu_e + \mu_h\right) \tag{S2}$$

Where is e the elementary charge constant, ε is the relative permittivity of the material, and ε_0 is the vacuum permittivity constant. So, the k_{L} of PBDTTT-E:$PC_{71}BM$ with DIO treated is 7.61´ 10^{-10} cm^3s^{-1}.The k_{L} of device without DIO is 6.17´ 10^{-10} cm^3s^{-1} .

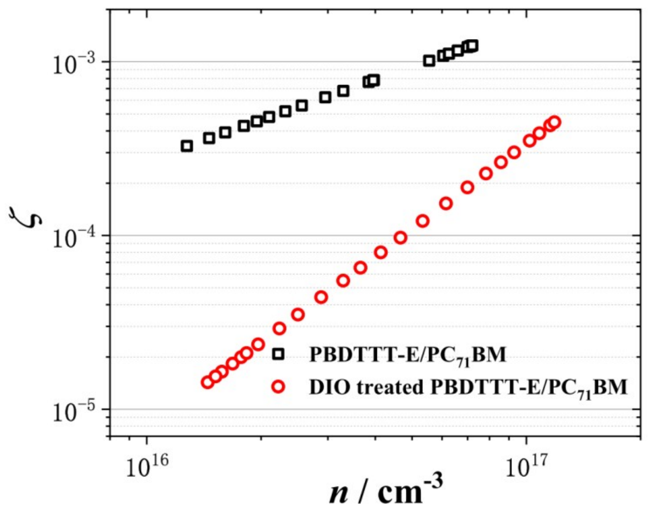

Figure S2: The langevin reduction factor (ζ) as a function of the charge carrier density (n). the black □ and red ○ represent the PBDTTT-E:$PC_{71}BM$ without and with DIO treated devices, respectively.

3. Extracted mobility of the hole and electron-only devices

Based on Figure 2, we choose the current density when V-V_{bi} near 4V as J_{SCL}, which is used to calculate the SCLC mobility. The method has been used and published in other groups [S2,S3].

Table S1. Extracted mobility of the hole and electron-only devices

Active layer	Hole Mobility ($cm^2V^{-1}S^{-1}$)	Electron Mobility ($cm^2V^{-1}S^{-1}$)
PBDTTT-E:$PC_{71}BM$	5.818×10^{-4}	4.425×10^{-4}
DIO PBDTTT-E:$PC_{71}BM$	4.910×10^{-4}	7.723×10^{-4}

In addition, we combined the Mott-Gurney equation and Frenkel effect [S4]:

$$J = \frac{9}{8}\varepsilon_0\varepsilon_r\mu_0\frac{(V - V_{bi})^2}{d^3}\exp(\beta_F\sqrt{\frac{V - V_{bi}}{d}}) \tag{1}$$

Here $E = \frac{V - V_{bi}}{d}$, We can get

$$J = \frac{9}{8}\varepsilon_0\varepsilon_r\mu_0\frac{E^2}{d}\exp(\beta_F\sqrt{E}) \tag{2}$$

The μ_0 is the zero field mobility, E is electric field, β_F is the field dependent parameter.

Take the natural logarithm on both sides of the equation (2):

$$\ln\left(\frac{J}{E^2}\right) = \ln(\frac{9}{8}\varepsilon_0\varepsilon_r\frac{\mu_0}{d}) + \beta_F\sqrt{E} \tag{3}$$

There is a linear relationship between ln(J/E^2) and $E^{1/2}$

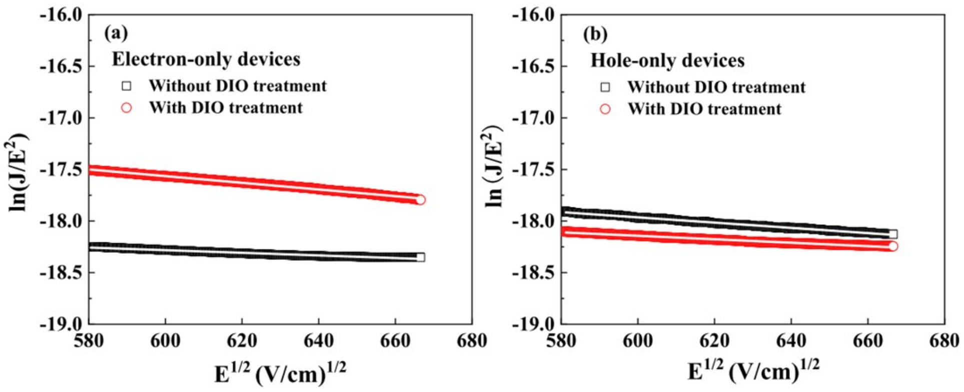

Figure S3: ln(J/E^2) as a function of high E$^{1/2}$ of electron-only devices (a) and hole-only devices (b) with (red○) and without DIO treatment (black □). The white lines are the linear fit lines followed equation (3).

The parameters extracted from the fit for the devices are summarized in Table S2, the negative Pool−Frenkel coefficient is consistent with P3HT:PCBM system.[S4]

Table S2. Parameters Extracted by the Fit for Devices

	Active layer	μ_0 ($cm^2V^{-1}S^{-1}$)	β_F $(cmV^{-1})^{1/2}$
Electron-only devices	Without DIO	7.40×10^{-4}	-0.00128
	With DIO	4.91×10^{-3}	-0.00324
Hole-only devices	Without DIO	2.18×10^{-3}	-0.00254
	With DIO	1.09×10^{-3}	-0.00168

According to Poole-Frenkel formula:

$$\mu(E) = \mu_0 \exp(\beta_F \sqrt{E}) \tag{4}$$

We can get the mobility(μ) as a function of applied electric field (E):

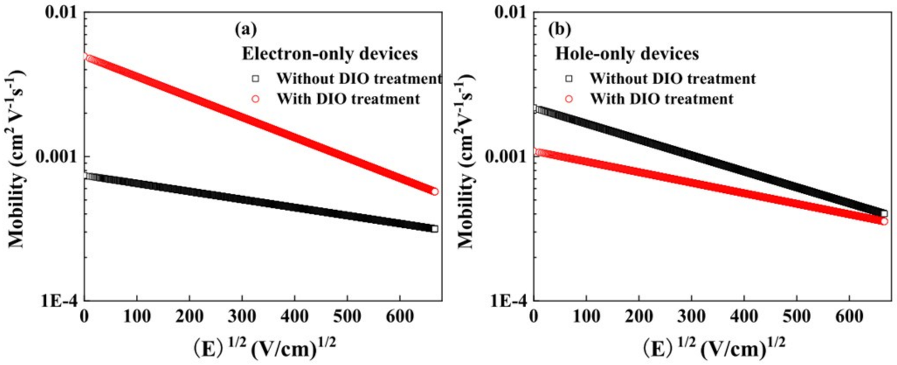

Figure S4: μ(E) as a function of $E^{1/2}$ of electron-only devices (a) and hole-only devices (b) with (red○) and without DIO treatment (black □).

[S1] M. C. Heiber, T. Okubo, S.-J. Ko, B. R. Luginbuhl, N. A. Ran, M. Wang, H. Wang, M. A. Uddin, H. Y. Woo, G. C. Bazan and T.-Q. Nguyen, *Energy Environ. Sci.,* 2018, **11**, 3019.

[S2] Y.-F. Zheng, S.-G. Li, D. Zheng, J.-S. Yu, *Organic Electronics*, 2014, **15**, 2647-2653.

[S3] Q.-C. Shang, J.-S. Yu, R. Hu,Z.-X. Liu, J. Cheng, Y. Li, X.-X. Shai, M.-M. Huo, X. Yang and L. Li, *ACS Appl. Mater. Interfaces,* 2020, **12**, 13051−13060.

[S4] N. Chandrasekaran, E. Gann, N. Jain, A. Kumar, S. Gopinathan, A. Sadhanala, R. H. Friend, A. Kumar, C. R. McNeill and D. Kabra, *ACS Applied Materials & Interfaces*, 2016, **8**, 20243–20250.
